# A Case of Ovariotomy

**Published:** 1853-01

**Authors:** S. D. Gross

**Affiliations:** Professor of Surgery in the University of Louisville


ART. III.—*A Case of Ovariotomy.* By S. D. GROSS, M. D.,
Professor of Surgery in the University of Louisville.

It has been justly observed, by an eminent writer, that the publication of unsuccessful cases is often more edifying and useful than that of favorable ones; because they serve as beacon lights to guide and direct the practitioner in his future movements. Although the subjoined instance exhibits no novelty, or peculiarity, in any of its features, and although every thing was done, apparently, that could be done, to save the patient, yet the details will not, it is believed, prove altogether uninteresting or uninstructive. It is proper to state that I had the kind advice, both at the operation and during the after treatment, of my friend, Dr. Joseph Knight, and of my colleague Professor Miller, the latter of whom, in particular, rendered me most valuable assistance during the excision of the tumor. My acknowledgments are also due to Dr. Cheneweth, Dr. Thomson, Dr. Henry, Dr. William H. Miller, and Professor Rogers.

Miss D., the subject of this case, was twenty-two years of age, rather tall and slender, and of nervo-sanguineous temperament. Her general health had always been pretty good until within a short time of the operation which I am about to describe; she had menstruated regularly since her fifteenth year, and had never suffered materially from derangement of her digestive organs. In the winter of 1848, she first perceived a small tumor in the lower part of her abdomen, free from pain and tenderness, perfectly movable and inclining somewhat more to the right side than to the left. This continued steadily to increase in size, at first gradually, and afterwards rather rapidly, until the following autumn, when it was so large as to cause serious inconvenience by its weight and bulk, as well as by its pressure upon the diaphragm and abdominal viscera. I saw Miss D. for the first time on the 11th of May, 1849, along with Dr. Knight and Professor Miller. Her general health was still good although she was considerably emaciated; but the abdomen was greatly distended by a tumor, which, upon examination, was found to be partly fluid and partly solid, smooth and uniform upon the surface, and entirely devoid of pain and tenderness. The uterus appeared to be perfectly sound, even the menstrual function had never been seriously deranged. But little was done in the way of constitutional treatment. On the 5th of June, with the concurrence of Dr. Knight and Dr. Miller, I introduced a trocar into the swelling and drew off three gallons of a thick, ropy, drab colored fluid, readily coagulated by heat, alcohol, and acids. The paracentisis was followed by great relief; but as the fluid soon began again to accumulate, it was thought best to advise the excision of the tumor, as the only chance of effecting a permanent cure. The patient promptly and cheerfully assented to our decision.

The operation was performed on the 19th of June. The bladder and bowels having been previously emptied, the patient was placed upon a table, and put fully under the influence of chloroform. The incision was made along the linea alba, and extended from near the pubes to three inches above the umbilicus, being at least one foot in length. The tumor being thus completely exposed, was found to be very red and vascular upon its surface, and to adhere all around, inferiorly, by bands of false membrane, which, however, were easily detached by the hand. The pedicle was quite narrow, and, after being surrounded by a stout ligature, it was divided and the morbid growth lifted from its bed. Although the ligature had been tied with great firmness, it immediately slipped off after the part was cut, thus rendering it necessary to apply another. Previously, however, to doing this a large artery included in the pedicle, and bleeding freely, was secured separately, lest the large ligature might come off again, and thus lead to serious, if not fatal hemorrhage. A fasciculus of false membrane, on the left side, appearing to bleed, was treated in a similar manner. The blood being now carefully removed from the peritoneal cavity, the ligatures were brought out at the inferior angle of the wound, the edges of which were next approximated by nine twisted sutures, the needles being carried through the muscular fibres, about an inch and a quarter from each other. Strips of ising-glass plaster were now placed in the intervals between the needles, and a narrow and rather thick compress was stretched along each side of the wound; the whole being supported by a broad bandage, passed round the abdomen and upper part of the thighs. The patient, pale, considerably exhausted, and in great pain, was put to bed, and took immediately one grain of morphia, followed by half a grain more in two hours.

The operation was performed at 11 o'clock in the morning, and at 4 o'clock in the afternoon I found my patient quite comfortable. She had slept a little, and was nearly free from pain; the skin was moist, the extremities warm, and the pulse soft and one hundred and twenty in the minute. She had vomited once soon after the operation, probably in consequence of having drunk too much water.

On the morning of the 20th, I learned that the patient had slept well nearly all night; she had urinated without difficulty; the abdomen was free from pain and distention; she could lie equally well on her back and sides. In the evening, the abdomen was slightly tumid and somewhat tender on pressure, the countenance was a little flushed, and the pulse was about 120. Half a grain of morphia was ordered to be taken at bed time.

On the 21st there was some tympanitis, but in other respects the patient was doing well. She had again passed a good night, had considerable appetite, and was free from abdominal tenderness.

On the 22d, an enema of half an ounce of castor-oil and a pint of flaxseed tea was ordered, and this was succeeded during the day by a slight fecal discharge, accompanied by a considerable quantity of flatus.

From this time on everything progressed favorably. On the 26th she took a mild vegetable pill, which was followed by two small alvine evacuations; on the 27th, eight days after the operation, I removed the four upper needles, the rest being allowed to remain until the next morning. The wound, which was now supported merely by broad adhesive strips, had united entirely by the first intention, except at the sutures, where slight ulceration had commenced; the bowels moved spontaneously; and everything promised a happy issue.

On the 2d of July, menstruation set in, and continued until the 4th, when it ceased. The patient at this time looked pale and feeble, she was accordingly directed to take quinine and brandy, in moderate quantities. Under this treatment, together with the aid of a light but nutritious diet, and good nursing, she went on well until about the 8th of July, when in consequence, apparently, of exposure to damp and heavy draughts of air, occasioned by a change in the weather, she was seized with a severe chill, followed by excessive prostration. Means were immediately resorted to for producing reaction, but several hours elapsed before she completely rallied, and she was never well afterwards. It was but too evident that peritonitis was going on, and that, notwithstanding the use of blisters and appropriate internal remedies, the case must terminate fatally. Miss D. lingered until the 17th of July, at 4 o'clock, when she expired.

The dissection made twelve hours after death, was conducted by my friend, Dr. T. G. Richardson, who has kindly furnished me the following account of it. There was no external sign of putrefaction; the body was greatly emaciated; the abdomen was tense and tympanitic, with a sense of obscure fluctuation, and upon puncturing it, a considerable amount of gas issued from it. The walls of the abdomen were so closely adherent to the viscera as to be dissected off with difficulty; and the parietal portion of the peritoneum, greatly thickened throughout its whole extent, was firmly attached to the omentum, liver, spleen and intestines, which were so completely buried in lymph as to be hardly distinguishable from each other. Owing to the peculiar manner in which the viscera were united to each other, the peritoneal cavity was divided into three distinct compartments, namely, two lateral and abdominal, and one pelvic, the former containing each about half a gallon of turbid serum, mixed with shreds and cakes of lymph, and the latter about a pint of sero-purulent matter.

The internal extremity of the ligature which had been cast round the pedicle of the tumor lay loose in the pelvic cavity, but so completely were the organs concealed by the inflammatory deposits that it was impossible to discover the point where it had been attached. The outer extremity was hanging out at the inferior angle of the wound, where it had effectually resisted the various attempts that had been made to separate it. The left ovary, with difficulty found, was somewhat softened, but in other respects perfectly natural. The uterus, with the exception of a little congestion of its lining membrane, was also unchanged.

The internal surface of the stomach and bowels, and the substance of the liver and spleen presented nothing of a pathological character. The bladder also was sound.

It will thus be seen that Miss D. died from the effects of peritonitis, the first open and well-marked symptoms of which became apparent only about nine days before death. The probability, however, is, judging from the enormous quantity of lymph that was found at the autopsy, and the matted condition of the abdominal and pelvic viscera, that it was present, in a latent form, at a much earlier stage of the case. Be this as it may, she survived the operation nearly one month, and there was, for the first eighteen days, every reason to believe that the case would have a favorable issue. That the cold which she took at the end of the first week in July exerted a powerful influence in bringing about an unfortunate result, cannot be doubted.

The tumor, after its removal, was found to be of an ovoidal shape, being larger above than below, soft and fluctuating, and of a uniform outline, except at three points, where it was rendered slightly protuberant by the presence of small cysts. It was from ten to twelve inches in length, about eight in breadth, and nearly the same in the antero-posterior diameter. Its weight was about nine pounds. Laid open, it was found to consist of a single sac, filled with reddish serum and grumous blood, adhering to its surface. Its walls were fully an inch in thickness, and bore a very close resemblance to those of the gravid uterus. The adventitious or accessory cysts, previously mentioned, were attached to the main sac, but did not communicate with it. The largest, situated at the base of the tumor, was of the size of a hen's egg, and was occupied by a substance similar to that of the main bag; while the two smaller were filled with sero-albuminous fluid.

